# The Role of Genetics in Moderating the Inter-Individual Differences in the Ergogenicity of Caffeine

**DOI:** 10.3390/nu10101352

**Published:** 2018-09-21

**Authors:** Kyle Southward, Kay Rutherfurd-Markwick, Claire Badenhorst, Ajmol Ali

**Affiliations:** 1 School of Sport, Exercise and Nutrition, Massey University, North Shore Mail Centre, Private Bag 102 904, Auckland 0745, New Zealand; K.A.Southward@massey.ac.nz (K.S.); C.Badenhorst@massey.ac.nz (C.B.); 2 School of Health Sciences, Massey University, Auckland 0745, New Zealand; K.J.Rutherfurd@massey.ac.nz; 3 Centre for Metabolic Health Research, Massey University, Auckland 0745, New Zealand

**Keywords:** CYP1A2, ADORA2A, time trial performance, caffeine metabolism, pharmacological ergogenic aid

## Abstract

Caffeine use is widespread among athletes following its removal from the World Anti-Doping Agency banned list, with approximately 75% of competitive athletes using caffeine. While literature supports that caffeine has a small positive ergogenic effect for most forms of sports and exercise, there exists a significant amount of inter-individual difference in the response to caffeine ingestion and the subsequent effect on exercise performance. In this narrative review, we discuss some of the potential mechanisms and focus on the role that genetics has in these differences. CYP1A2 and ADORA2A are two of the genes which are thought to have the largest impact on the ergogenicity of caffeine. CYP1A2 is responsible for the majority of the metabolism of caffeine, and ADORA2A has been linked to caffeine-induced anxiety. The effects of CYP1A2 and ADORA2A genes on responses to caffeine will be discussed in detail and an overview of the current literature will be presented. The role of these two genes may explain a large portion of the inter-individual variance reported by studies following caffeine ingestion. Elucidating the extent to which these genes moderate responses to caffeine during exercise will ensure caffeine supplementation programs can be tailored to individual athletes in order to maximize the potential ergogenic effect.

## 1. Introduction

Caffeine was placed on the World Anti-Doping Agency’s (WADA) banned list in 1984 and remained there until 2004 when it was removed from this list and placed on the monitoring program after it was determined that it no longer satisfied two of the three criteria needed to be on the banned list. Since the removal of caffeine from the banned list, the use of caffeine as an ergogenic aid amongst athletes has become widespread around the world with one study reporting approximately 73.8% of athletes will have consumed caffeine shortly before or during an event, with a higher prevalence in endurance athletes [1].

The first study to show the ergogenic potential of caffeine was published in 1907 [2], there was no further research in this area until the work of Ivy and Costill in the late 1970s [2,3,4]. Ivy and Costill [3,4] suggested that the ergogenic effect of caffeine was due to increased lipid mobilisation and utilisation through both direct action on fat stores as well as through stimulating the release of cortisol and norepinephrine, thus increasing lipolysis [5,6,7,8]. An increase in lipid mobilisation was hypothesised to increase glycogen sparing and thus, delayed fatigue during endurance exercise [3,4]. However, since then, other studies have shown that caffeine does not significantly increase lipid metabolism during exercise [9,10,11,12,13]. Evidence now indicates the ergogenicity of caffeine is most likely due to its effect as a potent adenosine receptor antagonist, whereby it blocks the actions of adenosine primarily in the brain [9,14,15].

The effects of adenosine on the central nervous system (CNS) have been well documented [16,17]. Adenosine has been shown to decrease feelings of arousal, alertness and vigilance, which increases central fatigue and negatively impacts on exercise performance [16,17]. Normally, adenosine slowly accumulates throughout the day, as well as during exercise when there is insufficient oxygen to regenerate adenosine triphosphate (ATP). Adenosine concentrations decrease during rest or sleep when ATP stores are regenerated [18,19]. Adenosine has been shown to down regulate various neurotransmitters such as dopamine, serotonin, glutamate, acetylcholine and norepinephrine [16,17]. Dopamine is a key neurotransmitter in parts of the brain which regulates behavioral activation and effort-based behavioral processes; thus, decreases in dopamine concentrations can lead to a reduced effort during exercise and a reduction in overall exercise performance [20]. This is more evident in endurance exercise where central fatigue plays an important role in moderating exercise performance, compared to strength and speed-based exercise such as sprinting, where peripheral fatigue may have a larger impact on overall performance.

The effects of adenosine are inhibited through competition by caffeine at the adenosine receptor sites. Caffeine, as well as theophylline and paraxanthine, caffeine metabolites, have a similar structure to adenosine, which allows them to bind to adenosine receptors throughout the body. It is the antagonism of adenosine by caffeine and theophylline molecules which likely has the biggest impact on the ergogenicity of caffeine, particularly during endurance exercise by reducing the effects of adenosine and ultimately decreasing feelings of tiredness and improving vigilance, arousal and a willingness to exert effort during exercise [15,16,21].

Despite well-documented overall improvements in exercise performance following caffeine ingestion [9,15,22,23], there exists a significant variation, both between individuals and between studies, in the responses to caffeine ingestion [24]. Studies which have reported individual data have shown that a number of individuals either do not respond to caffeine supplementation, such that their performance is unchanged between placebo and caffeine trials, or performance is decreased following caffeine supplementation (Table 1), as opposed to the majority of individuals who show improved exercise performance following caffeine ingestion. These studies show that approximately 33% of individuals in these investigations did not improve their endurance time-trial performance following caffeine ingestion. While this shows evidence of the variance between individuals and studies, the cause of the variance is not yet fully understood. A recent publication by Pickering and Kiely [25], provided some discussion around the inter-individual variation in the ergogenicity of caffeine, however, the present paper looks to expand upon this further with specific reference to endurance sports as well as taking a more in-depth look at the genes associated with caffeine ergogenicity.

## 2. The Effect of CYP1A2 on Inter-Individual Differences in Ergogenicity of Caffeine

The response to caffeine and the potency of the effects of caffeine seem to be multifaceted. Caffeine habituation [7,45], metabolism of caffeine [9,46], method of caffeine ingestion [47], caffeine dosage [9,48], training status [49,50], and timing of caffeine ingestion [41] have all been identified as having an effect on the ergogenicity of caffeine. Similarly, oral contraceptives, pregnancy, ethnicity, age, and smoking have all been suggested to affect the metabolism of caffeine [51]. Genetic variation in specific genes, namely CYP1A2 (rs762551) and ADORA2A (rs5751876), have also been suggested to have a significant effect on the responses to caffeine ingestion and the metabolism of caffeine [51].

The cytochrome P450s (CYP) are a family of haemoprotein enzymes which are responsible for approximately 75% of all drug metabolism [52]. The cytochrome P450 family 1 subfamily A member 2 (CYP1A2) is predominantly found in the liver and metabolises many clinical drugs and endogenous compounds [53]. The CYP1A2 enzyme is responsible for >90% of caffeine metabolism, breaking it down into the three metabolites: paraxanthine (81.5%), theobromine (10%) and theophylline (5.4%), while CYP2E1 is responsible for the majority of the transformation between caffeine to theophylline and theobromine [54,55].

As the majority of caffeine metabolism is determined by CYP1A2 enzyme activity, variations to the gene encoding for the CYP1A2 enzyme will alter its inducibility, thus significantly impacting the metabolism of caffeine. A single nucleotide polymorphism (−163 C > A) at position 734 within intron 1 (rs762551) has been identified as the major source of inducibility of CYP1A2 and thus caffeine metabolism [46,51,53]. Individuals with the homozygous A/A allele show enhanced caffeine metabolism and have been classified as “fast metabolisers”, whereas C allele carriers (A/C and C/C) have a reduced caffeine metabolism and are known as “slow metabolisers” [51,53]. Therefore, slow metabolisers are likely to have a prolonged caffeine half-life compared to fast metabolisers. Half-lives of paraxanthine, theobromine and theophylline are ~3 h, ~6 h, and ~7 h respectively [56]. On average, 40% of the general population carry the A/A genotype while 50% and 10% carry the A/C and C/C genotypes, respectively [36,46]. A slower caffeine metabolism would seem to be beneficial for endurance performance as the effects of caffeine would be longer lasting as well as potentially more pronounced, however, some studies have reported the opposite effect [36,44].

Relative to the amount of research on caffeine and performance *per se*, there is limited information on the effects of CYP1A2 genotypes on the ergogenicity of caffeine (Table 2). Two studies found greater performance improvements in individuals with the A/A genotypes compared to C allele carriers [36,44]; two studies found no differences between genotypes in time-trial performance [57,58]; and one study found individuals with the A/C genotype performed better in a 3 km time-trial compared to individuals with the A/A genotype [59]. Other studies [60,61,62] found no effect of CYP1A2 genotype on the ergogenicity of caffeine using their respective protocols.

It is evident that the results of investigations into the effects of CYP1A2 genotypes on the ergogenicity of caffeine remain equivocal. This may be largely due to the protocols used by the majority of these studies and in some instances the protocol may be counter-productive to the aim of the study. Caffeine as an ergogenic aid has been shown to be most effective and reliable when used for endurance exercise [63], therefore, using a short duration protocol such as the 30 s Wingate test or sport-specific skill tests will inherently lead to less reliable results. Using a long duration endurance protocol to investigate the effects of CYP1A2 on the ergogenicity of caffeine would be beneficial as it would allow for larger changes to be seen in caffeine metabolism between fast and slow metabolizers as caffeine would have had more time to breakdown within the body. It would therefore be more specific and applicable to the endurance sports it is most commonly consumed in, thus having a greater impact. Caffeine is most commonly consumed for endurance sports such as triathlons and marathons [1] which typically last between 1–3 h for half-marathons, marathons, sprint and standard distance triathlons, and between 4–15 h for half and full iron-man events. Therefore, while exercise protocols which last longer than 5 min could technically be considered endurance exercise, endurance events usually last significantly longer than many of the protocols used in the current literature. The protocols used to examine the ergogenicity of caffeine on endurance exercise in future studies should attempt to use exercise protocols lasting longer than 1 h.

Furthermore, protocols used for a novel area of research, such as the effects of genetics on the ergogenicity of caffeine, should be controlled to provide the best chance of obtaining a favorable outcome as proof of concept. Using a short duration test (strength or power tests), or sport-specific skill tests, which have been shown to produce less reliable improvements following caffeine ingestion [63], are unlikely to provide conclusive results when examining the effect of CYP1A2 genotypes on the ergogenicity of caffeine using these protocols. Conversely, endurance exercise protocols have shown much more reliable results compared to short duration exercises and would aid in isolating the effect of CYP1A2, as well as other genes, on the ergogenicity of caffeine. However, well-designed and well-controlled studies using shorter duration protocols should still be conducted as there are many sports, which do not last longer than 1 h, where caffeine may still have an ergogenic effect.

Of the studies included in Table 2, only those by Algrain [57] and Giersch [58] measured caffeine pharmacokinetics and in both cases found no difference in caffeine metabolism between individuals with the A/A allele and C allele carriers. However, Giersch et al. [58] measured caffeine metabolites for 1 h post caffeine ingestion (6 mg·kg^−1^ anhydrous caffeine pill), and Algrain et al. [57] recorded caffeine metabolites for 65 min post ingestion (300 mg caffeinated gum). When using anhydrous caffeine in pill form, peak caffeine concentration usually occurs around 1 h post ingestion [9] and around 30 min after using caffeinated gum [64]. Therefore, measuring caffeine metabolites for 1 h post-caffeine ingestion (via a capsule) would mostly measure caffeine absorption, which is not determined by CYP1A2, rather than metabolism. Thus, studies investigating the effects of CYP1A2 on the ergogenicity of caffeine should focus on using protocols which last >1 h, which allows caffeine metabolism to be measured over a 2-h period should caffeine be administered 1 h prior to exercise.

While it is expected that the CYP1A2 genotype affects caffeine metabolism, some studies have reported no difference in the rate of caffeine metabolism between fast and slow metabolizers in healthy adults [46,57,58]. CYP1A2 genotype affects the inducibility of the CYP1A2 enzyme, which results in changes to the metabolism of caffeine. Individuals with the A/A allele have a higher CYP1A2 inducibility compared to the A/C and C/C alleles [46,51]. The results of these studies may be partly explained by environmental factors which have also been shown to affect the inducibility of CYP1A2, including consumption of cruciferous foods (broccoli and Brussels sprouts), heavy exercise, tobacco smoke, oral contraceptives and various medicines (fluvoxamine, omeprazole) [65]. Combining multiple factors could lead to a greatly increased or reduced activity of CYP1A2, potentially up to 60-fold [65]. Therefore, individuals with the A/C or C/C CYP1A2 allele may have a similar caffeine metabolism to an individual with the A/A allele due to increased inducibility from environmental factors. These factors should be kept in mind when examining studies which have reported effects of CYP1A2 genotype on caffeine ergogenicity and metabolism, and future studies should control or record the use of potential potent inducers and inhibitors of CYP1A2 during caffeine trials.

While the current literature is equivocal as to whether CYP1A2 has an effect on the ergogenicity of caffeine, it is likely that it still has a role in mediating the effects of caffeine on exercise performance. The effects of CYP1A2 on the ergogenicity of caffeine may be more evident in endurance events lasting longer than 1 h, where the metabolism of caffeine may have a more pronounced effect, as those who metabolize caffeine faster would not maintain high circulating levels of caffeine throughout the event compared to those with a slower metabolism of caffeine, unless further ingestion occurred. It should be noted that the half-life of caffeine is 2–12 h and on average 4–6 h [66] in most adults; and it is not yet known to what degree caffeine metabolism is altered between fast and slow metabolizers. Therefore, it is unknown at what time point there would be a large enough difference in the circulating levels of caffeine between fast and slow metabolizers to have a significant impact on the ergogenicity of caffeine.

It has been suggested that fast metabolizers may receive a greater ergogenic effect from caffeine due to the faster metabolism of caffeine into the metabolites, theophylline and paraxanthine [37]. While it has been shown that the concentrations of the metabolites are likely too small to have an ergogenic effect [67], it should be noted that paraxanthine and theophylline are both adenosine antagonists as well [9]. Although the concentrations of paraxanthine and theophylline may be too small in isolation, together with caffeine, they may still provide a noticeable ergogenic effect. The effects of the CYP1A2 genotype on caffeine ergogenicity may be dependent on the duration of exercise, where longer duration exercise (>1 h) may be more suited to slow metabolizers and short-term high intensity exercise may be more suited to faster metabolizers of caffeine. However, this may be highly dependent on the difference between metabolism rates which currently have not been thoroughly explored and reported. In a letter to the editor, Pickering [68] echoed similar sentiments and stated that while several recent studies have found C allele carriers were not reported to have an ergogenic effect from caffeine, it may be due to the time at which caffeine was ingested, advocating for an earlier time of caffeine ingestion to test this hypothesis. Furthermore, studies should record and report caffeine pharmacokinetics before, during and after exercise when investigating CYP1A2 genotypes on caffeine ergogenicity in order to determine the magnitude of the effect CYP1A2 has on the metabolism of caffeine.

## 3. The Effect of ADORA2A on Inter-Individual Differences in Ergogenicity of Caffeine

The ADORA2A (C→T) gene encodes for the adenosine receptor A2A found predominantly in the brain and has a role in the down-regulation of dopamine and glutamate release [16,17]. Genotype distributions are varied between studies, however, approximately 45% of people carry the C/T allele while the T/T and C/C carriers range between 20–30% [69,70].

A number of studies have investigated the effects of ADORA2A gene variation on responses to caffeine ingestion [69,71,72,73,74,75]. The studies by Alsene et al. [75] and Childs et al. [74] both reported greater increases in self-reported caffeine-induced anxiety in individuals with the T/T allele compared to those with the C alleles. However, Retey et al., [71] reported a greater proportion of C/C genotype in individuals who rated themselves as caffeine “sensitive”, and a greater prevalence of T/T genotype in individuals who rated themselves as caffeine “insensitive”. The ADORA2A genotype may also affect habitual caffeine intake, as a study reported ADORA2A knockout mice self-administered less caffeinated solution compared to wild-type mice [76]. This suggests that ADORA2A may have a regulating role in the appetitive properties of caffeine, which is likely to influence habitual caffeine intake. A later study in humans [69] reported that CYP1A2 genotype had no effect on caffeine intake, however, individuals with the ADORA2A T/T allele had lower habitual caffeine consumption compared with the C allele carriers. This would suggest that individuals with the T/T genotype may have reduced habitual self-administered caffeine due to negative feedback. This supports the work of others [74,75] who reported greater caffeine-induced anxiety in T/T genotypes compared to C/C genotypes. A recent study [73] reported an increase in caffeine-induced anxiety in individuals with the T/T genotypes but not the C/T and C/C genotypes [73]. However, these results were mediated by habitual caffeine consumption, as caffeine-induced anxiety was greater in low and non-users of caffeine compared to medium and high users of caffeine. Caffeine-induced anxiety and habitual caffeine consumption are important factors which play a role in the ergogenic effects of caffeine, both of which have been associated with ADORA2A polymorphisms. Individuals with high or very low caffeine-induced anxiety, due to ADORA2A genotype may experience ergolytic or no ergogenic effects from caffeine consumption, respectively. Similarly, ADORA2A may influence habitual caffeine consumption which may attenuate the ergogenic effect of caffeine which is important for caffeine supplementation in days prior to competitions.

Homodimerisation of ADORA1A and ADORA2A has also been suggested to affect caffeine concentration as the binding of caffeine to the ADORA1A and ADORA2A receptors increases the affinity for a second caffeine molecule to bind to the ADORA1A dimer [77]. The increased uptake in caffeine by the adenosine receptors potentially leads to a decrease in local caffeine concentration and may partially explain the biphasic effects of caffeine on locomotors activation. After caffeine has binded to the adenosine receptor dimer it increases the affinity of adenosine and caffeine molecules to bind to the remaining receptor. Therefore, at low doses of caffeine (~<2 mg·kg^−1^) there is not sufficient caffeine to bind to both adenosine receptors of the dimer, thus the effects of caffeine are attenuated due to adenosine binding to the remaining receptor site. However, at high doses of caffeine, there is sufficient caffeine that both adenosine receptor sites can be occupied by caffeine rather than a caffeine and adenosine molecule.

To the authors’ knowledge, only one study [78] has investigated the effects of ADORA2A genotype on exercise performance following caffeine ingestion. Loy et al. [78] examined the effects of ADORA2A genotype on caffeine ergogenicity during a 30 min cycle in a randomized cross-over design. Twelve females performed 20 min of moderate-intensity cycling followed by a maximal 10 min cycle time-trial. Results of the 10 min time-trial revealed only one individual with the C/T and C/C group (*n* = 6) improved time-trial cycle performance following caffeine ingestion, whereas all participants in the T/T group (*n* = 6) showed improvements following caffeine ingestion. This is interesting as the T/T genotype has previously been associated with increased anxiety following caffeine ingestion [73,74,75]. While anxiety is generally thought to be ergolytic, it has not been shown to have a strong relationship with exercise performance [79]. Therefore, individuals with the ADORA2A T/T genotype may be more sensitive to the effects of caffeine as caffeine-induced anxiety may also be perceived as increasing arousal leading to potential ergogenic effects. Further research should be conducted using similar protocols to verify the results found by Loy et al. [78]. Moreover, different exercise modalities, as well as the use of perceptual and mood measures, should be utilised to determine the effects of ADORA2A genotype on the ergogenicity of caffeine during various forms of sports and exercise and any potential contributing mechanisms.

While CYP1A2 and ADORA2A remain the most researched genes with regards to the ergogenicity of caffeine, AHR (aryl hydrocarbon receptor) has been shown to affect caffeine metabolism through detecting polycyclic hydrocarbons such as those found in roasted coffee and induces the transcription of CYP1A2 in the liver [69,72,80,81]. While no studies have investigated the effects of AHR on the ergogenicity of caffeine, studies investigating the effects of CYP1A2 on the ergogenicity of caffeine should include AHR genotyping to elucidate the role it may have in caffeine metabolism. Together AHR, CYP1A2 and ADORA2A have all been associated with caffeine consumption. The variability of CYP1A2 and AHR has been associated with a 42% increase in coffee intake and a cooperative action between these two genes may exist for moderating caffeine consumption [82]. This adds support to the hypothesis that “individuals adjust their dietary caffeine consumption to maintain biological exposure levels of caffeine that elicit optimal stimulant effects” [83]. ADORA1A has also been reported to be largely responsible for the anxiogenic effects of caffeine as well as influencing the disruptions to sleep following caffeine ingestion [73]. Thus, ADORA1A could be another potential gene to investigate with caffeine supplementation, particularly for its disruptions to sleep and increased anxiety prior to and during competitions [84].

ADORA2A and CYP1A2 may also lead to unique interaction effects in response to caffeine ingestion. For example, an individual with the ADORA2A T/T and CYP1A2 C/C genotypes might have a greater ergogenic effect from caffeine when competing in a longer duration event (>1 h) as the slow metabolism may be beneficial in maintaining biologically active levels of caffeine in the body. Furthermore, an individual with ADORA2A T/T and CYP1A2 A/A may have a greater ergogenic benefit during a high-intensity, short-duration activity (such as a sprinting) through increased arousal and not requiring high levels of circulating caffeine for long periods of time. Therefore, individuals may require different levels of circulating caffeine to receive an equitable effect based on their genetics. Similarly, individuals will require more or less frequent consumption of caffeine to maintain their optimal circulating level of caffeine and respective ergogenic effect based on their genetics. However, factors such as habitual caffeine consumption, additional caffeine intake during the event, and individual reactions to caffeine may yet further influence the overall ergogenic effect. Further research is needed to examine and quantify the effect genetics may have on the ergogenicity of caffeine, and potentially identify any other genes associated with responses to caffeine ingestion.

## 4. Limitations and Future Considerations

The key limitation to this area of research is the relatively few number of studies investigating the effects of CYP1A2 and ADORA2A genotype on the ergogenicity of caffeine. There is a greater need to verify results of published studies rather than adding novel elements to the very small pool of studies currently available. This will be more useful to the end users such as coaches and athletes who need to know what the ergogenic effects of caffeine are likely to be, based on their particular combination of CYP1A2, AHR, ADORA2A, and ADORA1A genotypes. Therefore, studies should test participants for ADORA2A, ADORA1A, AHR and CYP1A2 genotypes as the combination of these genes may partly explain the equivocal results when investigating the ergogenicity of caffeine. Additionally, future studies should record caffeine and caffeine metabolite pharmacokinetics when administering caffeine and testing for CYP1A2 genotypes to gain a greater understanding of the differences between fast and slow metabolizers. This would also provide evidence for differences in caffeine metabolism for CYP1A2 genotypes which is the main premise for studies investigating the effects of CYP1A2 on the ergogenicity of caffeine. Studies investigating the effects of CYP1A2 on the ergogenicity of caffeine and caffeine metabolism should use longer duration protocols which last more than 1 h to ensure that differences between fast and slow metabolizers can more easily be determined. Currently, it cannot be stated conclusively whether fast or slow metabolizers perform better following caffeine ingestion because their different metabolism rates have not been measured and may in fact be similar between these individuals.

## 5. Conclusions

It is clear that various factors affect the ergogenic effects of caffeine and contribute to inter-individual variability in response to caffeine ingestion. Even with limited research, it appears that genetics plays a key role. Future research should further investigate which genes may affect the ergogenicity of caffeine as well as the mechanisms by which it is achieved. This would enable practitioners and coaches to tailor individualised caffeine supplementation regimes for athletes to achieve the maximum possible ergogenic effect in their specific sport.

## Figures and Tables

**Table 1:** Time-trial studies investigating ergogenicity of caffeine which reported individual data.

Study	Caffeine Dose	Number of Individuals Who Performed Worse in Caffeine Trials Compared to Placebo
Acker-Hewitt et al. [26]	6 mg·kg^−1^	2/10
Astorino et al. [27]	5 mg·kg^−1^	3/16
Astorino et al. [28]	5 mg·kg^−1^	1/9
Beaumont & James [29]	6 mg·kg^−1^	1/8
Christensen et al. [30]	3 mg·kg^−1^	4/12
Church et al. [31]	3 mg·kg^−1^	8/20
Desbrow et al. [32]	3 mg·kg^−1^	3/9
Desbrow et al. [33]	6 mg·kg^−1^	4/16
De Souza Goncalves et al. [34]	6 mg·kg^−1^	6/40
Graham-Paulson et al. [35]	4 mg·kg^−1^	1/11
Guest et al. [36]	2 mg·kg^−1^	38/101 ^1^
Guest et al. [36]	4 mg·kg^−1^	32/101 ^1^
O’Rourke et al. [37]	5 mg·kg^−1^	3/30
Pitchford et al. [38]	3 mg·kg^−1^	2/9
Roelands et al. [39]	6 mg·kg^−1^	4/8
Santos et al. [40]	5 mg·kg^−1^	2/8
Skinner et al. [41]	6 mg·kg^−1^	1/14
Stadheim et al. [42]	6 mg·kg^−1^	2/10
Stadheim et al. [43]	4.5 mg·kg^−1^	4/13
Womack et al. [44]	6 mg·kg^−1^	3/35
Total		124/379 (33%)

^1^ Same group of participants.

**Table 2:** Studies investigating the effects of CYP1A2 genotype on time-trial performance following caffeine ingestion.

Study	Sample	Caffeine Dose and Timing Prior to Exercise	Protocol	Results
Algrain et al. [57]	13 male and 7 female recreational cyclists	300 mg caffeinated chewing gum; 10 min	15 min@70% VO_2max_ followed by 10 min rest and 15 min performance cycle ride	No effect of genotype on performance ride performance
Giersch et al. [58]	20 male cyclists	6 mg·kg^−1^; 60 min	3 km TT cycle	No effect of genotype on 3 km TT performance
Guest et al. [36]	101 male competitive cyclists	2 mg·kg^−1^; 75 min	10 km TT cycle	Improved A/A genotype performance by 4.8%; No significant difference in A/C and C/C genotypes
Guest et al. [36]	101 male competitive cyclists	4 mg·kg^−1^; 75 min	10 km TT cycle	Improved A/A genotype 10 km TT performance 6.8%; Decreased C/C genotype 10 km TT performance by 13.7%
Klein et al. [60]	8 male and 8 female tennis players	6 mg·kg^−1^; 60 min	30 min intermittent treadmill running followed by tennis skill test	No effect of genotypes on tennis skill test
Pataky et al. [59]	25 male and 13 females	6 mg·kg^−1^; 60 min; 25 mL 1.14% caffeinated mouth rinse	3 km TT cycle	Greater improvements in 3 km TT in A/C genotypes compared to A/A genotypes
Puente et al. [62]	10 males and 9 female elite basketball players	3 mg·kg^−1^; 60 min	10 repetitions of: Abalakov jump test and change of direction and acceleration test; 20 min simulated basketball game	No effect of genotype on tests performance
Salinero et al. [61]	14 male and 7 females recreationally active	3 mg·kg^−1^; 60 min	30 s Wingate test	No effect of genotypes on Wingate performance
Womack et al. [44]	35 recreationally competitive male cyclists	6 mg·kg^−1^; 60 min	40 km TT cycle	Improved cycling TT performance to a greater degree in A/A genotypes compared to C allele carriers

TT: Time-trial.
